# Bilateral lower extremity amputation in a transgender female on estrogen therapy suffering from recurrent, medication-resistant arterial thrombi

**DOI:** 10.1016/j.jvscit.2024.101655

**Published:** 2024-10-22

**Authors:** Mitchell C. McDaniels, Patrick D. Conroy, Philip M. Batista

**Affiliations:** Department of Vascular and Endovascular Surgery, Cooper University Hospital, Camden, NJ

**Keywords:** Acute limb ischemia, Arterial thrombi, Estrogen therapy, Gender-affirming hormone therapy (GAHT), Transgender

## Abstract

This case report presents a 40-year-old transgender female with a history of gender-affirming hormone therapy who experienced recurrent, medication-resistant arterial thrombi leading to bilateral lower extremity amputations. Despite multiple endovascular and surgical interventions, including bypass grafting and catheter-directed thrombolysis, the patient developed recurrent thrombotic events even while on anticoagulation therapy. Hematologic evaluation for coagulopathy was unremarkable. The case underscores the need for greater understanding of gender-affirming hormone therapy’s long-term cardiovascular effects while highlighting the challenges in managing arterial thrombosis in transgender patients. Further research is required to guide optimal anticoagulation strategies in this population.

There are over 1.6 million individuals in the United States who identify as transgender today, compared with only 700,000 in 2011.^1^^,^^2^ This rapid growth has resulted in increased need and utilization of gender-affirming hormone therapy and surgery, despite no guidelines defining the standard of care across the lifespan.^2^^,^^3^ The cardiovascular implications of gender-affirming hormone therapy are still largely undefined. Previous literature has identified hormonal factors that may predispose patients to a hypercoagulable state and thrombus formation.^2^, ^3^, ^4^ Estrogen can alter platelet activation, platelet aggregation, and coagulation; thus, the long-term gender-affirming hormone therapy can cause potentially detrimental effects in this population resulting in loss of life or limb. This case report underscores the challenges of persistent and recurrent arterial thrombus in a transgender female and the need for a stronger understanding of the implications and management of peripheral artery disease in patients undergoing gender-affirming hormone therapy.

## Case presentation

A 40-year-old transgender female with past medical history of gender-affirming estrogen therapy for 5 years, 19.00 pack/years of smoking, post-traumatic stress disorder, and schizoid personality disorder, presents to our institution with 3 days of right lower extremity pain, several days of associated right foot edema and erythema, and hypoesthesia and discomfort throughout the leg progressing for months. She underwent angiogram and stenting of her right popliteal artery a year and a half ago at an outside facility, with inaccessible records, but the patient reports this intervention was to attend to “leg muscle cramping.” On exam by the vascular surgery team, the right lower extremity was noted to be red with subjective numbness and intact motor function. Right lower extremity dorsalis pedis and posterior tibial artery signals were absent, though contralateral signals were noted to be multiphasic. Arterial duplex of the right lower extremity showed triphasic waveforms down the level of the superficial femoral artery with occlusions of the popliteal stent and the anterior and posterior tibial arteries. Computed tomography runoff demonstrated occlusion of the right popliteal artery and stent with no distal reconstitution and minimal overall atherosclerotic disease.

**Fig 1:**
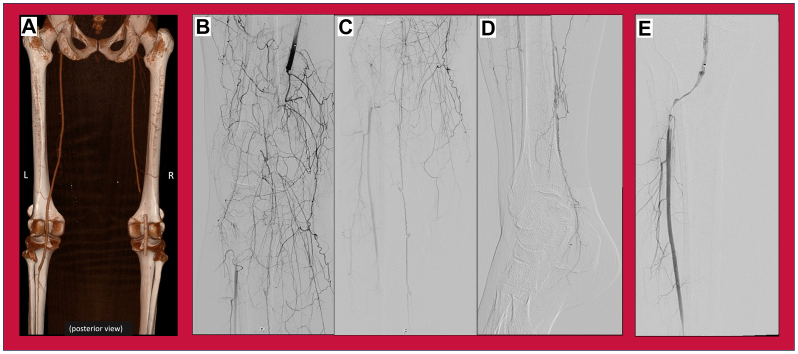
Initial computed tomography angiography (CTA) and angiogram, pre- and post-thrombolysis, during the index encounter for right acute limb ischemia. **(A)** Reconstructed three-dimensional CTA runoff posterior view showing occluded right popliteal artery proximal to stent; **(B-D)** Pre-intervention angiogram of the right leg; **(E)** post-thrombolysis angiogram of the right leg.

The patient was diagnosed with acute limb ischemia, and the decision was made to urgently intervene. Initial attempts at endovascular salvage successfully recanalized the popliteal stent, but no inline flow to the foot was established (Fig 1), despite days of thrombolytic therapy and multiple thrombectomies. She ultimately required right superficial femoral artery to dorsalis pedis artery bypass with great saphenous vein graft. An initial runoff was completed postoperatively, which showed no inflow disease, three vessel runoff past the ankle, and technically good anastomoses. The patient was anticoagulated with an unfractionated heparin drip titrated to a heparin anti-Xa level of 0.3 U/mL to 0.7 U/mL and was within goal throughout the early postoperative period. Despite these efforts, the bypass was found to be thrombosed 12 hours into the postoperative period, as seen on point-of-care ultrasound, which demonstrated no flow on color Doppler and associated intra-bypass echogenicity consistent with thrombus formation despite postoperative heparin. The patient remained without sensorimotor function, and the foot was cool on examination. Due to the patient’s persistent arteriothrombosis, further interventions were deemed futile, and the decision was made to proceed with below-knee amputation of the right lower extremity with ligation of the bypass graft at the level of the stump in an effort to maintain as much functionality of the young patient’s limb as possible. Due to the amount of swelling, this was a high risk below-knee amputation, and the risks and benefits of this procedure were discussed with the patient extensively, employing the use of shared decision-making.

Due to lack of known chronic disease, hematology was consulted for extensive coagulopathic investigation including beta-2-glycoprotein antibody, anti-cardiolipin antibody, homocysteine, antithrombin III, lupus-anticoagulant, Factor V Leiden, Prothrombin gene mutation, diluted Russell viper venom time mix and confirmation, Proteins C and S activities, and JAK2 V617 F mutation, all of which were unremarkable. Gender-affirming hormone therapy was ceased immediately, and she was discharged on a home regimen of aspirin and apixaban.

Two days after discharge, she presented for uncontrolled pain and multifocal areas of necrosis at the stump incision. She expressed difficulty attaining apixaban after discharge and had not yet initiated outpatient anticoagulant therapy. The stump continued to decompensate, with persistent leukocytosis and worsening wound dehiscence. Computed tomography angiography revealed occluded native superficial femoral artery and bypass graft, with patent profunda femoris artery. Due to the patient’s persistent intractable pain and inability to heal wounds, the decision was made to proximalize the amputation to above-knee amputation and excise the bypass graft. The patient was discharged on apixaban and aspirin.

Two months later, the patient was admitted for contralateral (left) limb ischemia with two weeks of tingling, severe pain, and a mottled, blue appearance. Of note, the patient had not had prior intervention on this limb. The patient reported adherence to apixaban and aspirin regimen. Pulses were absent to the level of the femoral artery. Motor function was intact, with sensory deficits to the plantar surface of the foot.

**Fig 2:**
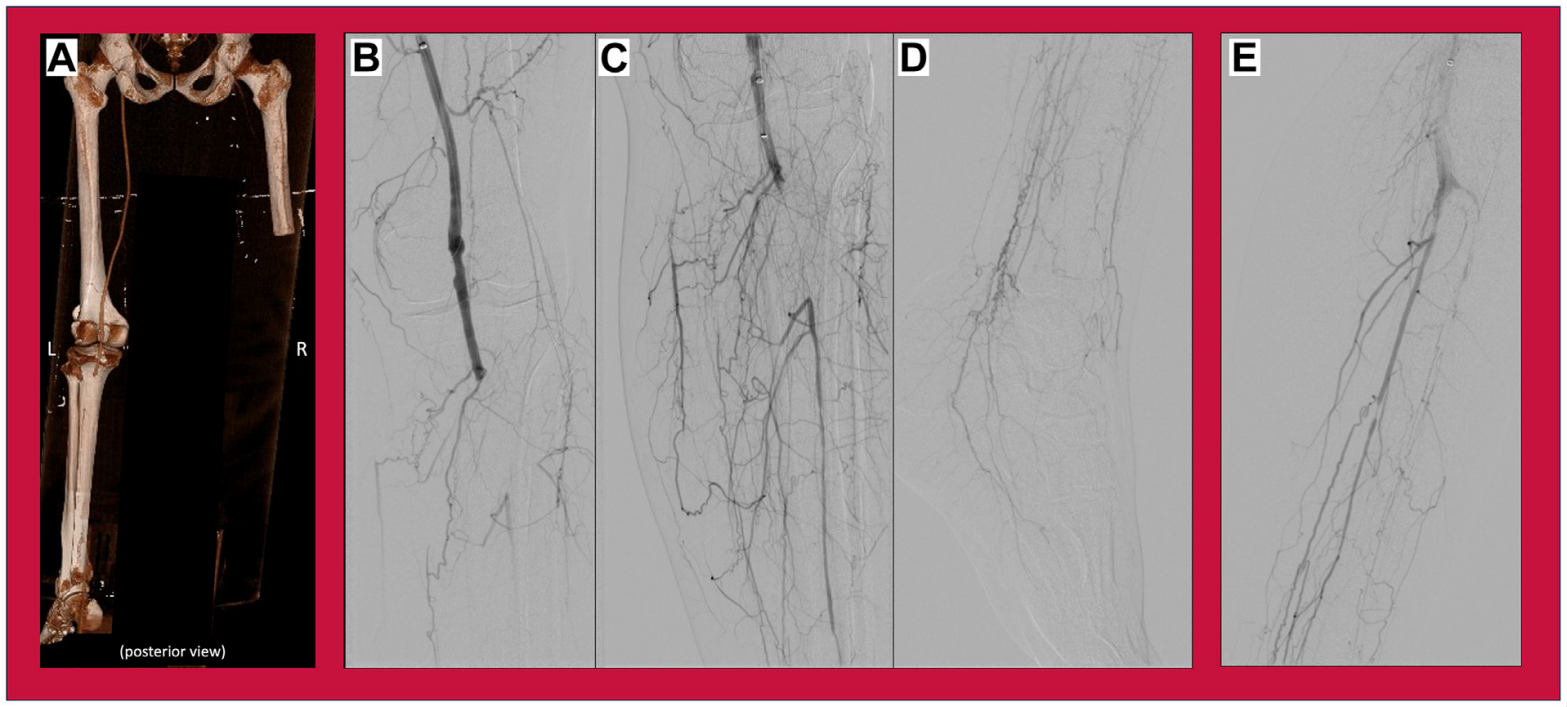
Computed tomography angiography (CTA) and angiogram, pre- and post-thrombectomy, during the third encounter, for left (contralateral to prior) acute limb ischemia. **(A)** Reconstructed three-dimensional CTA runoff posterior view showing occluded left popliteal artery proximal to stent; **(B-D)** Pre-intervention angiogram of the left leg; **(E)** Post-thrombectomy angiogram of the left leg.

Computed tomography angiography revealed occlusions of the left popliteal artery, posterior tibial, anterior tibial, and peroneal arteries, ultimately diagnosed as Rutherford Class IIA (Fig 2). Endovascular approach using a Penumbra device was unsuccessful in providing acceptable three-vessel runoff; thus, the procedure was converted to open thrombectomy with four compartment fasciotomies.

The patient recovered appropriately, maintaining patent vasculature. Given the persistence of her thrombus formation while adherent to an apixaban regimen, hematology recommended a change in her anticoagulation plan to rivaroxaban with aspirin. During this hospitalization, transthoracic echocardiogram with bubble study was completed and showed no signs of a patent foramen ovale, with low-normal ejection fraction. Also, computed tomography angiography of the chest, abdomen, and pelvis was done to evaluate for any atheroembolic source, all of which was negative.

Five months from index hospitalization, the patient presented with another episode of acute limb ischemia of the left lower extremity. All below-knee arteries were again found to be occluded, and catheter-directed thrombolysis was initiated. Three days after intervention, lysis check and angiogram demonstrated patent peroneal artery to the foot. Strong anterior tibial and posterior tibial signals were present at the conclusion of the case. Given yet another episode of thromboembolism, while compliant with rivaroxaban and aspirin therapy, therapeutic warfarin was initiated (international normalized ratio [INR] = 2-3) due to concern for insufficient direct oral anticoagulant response. Clopidogrel was initiated to optimize antiplatelet activity.

Five days following her most recent discharge, the patient returned for pain, numbness, and erythema of the left leg despite strict compliance with her medication regimen. Computed tomography angiography indicated unchanged arterial flow with peroneal-dominant runoff and no evidence of acute thrombosis. Due to intractable pain in the left lower extremity despite pain management consultation, the patient expressed her desire to undergo left lower extremity amputation after months of unremitting discomfort, citing the relief achieved from her previous contralateral above-knee amputation. Following formal capacity evaluation, the patient underwent left below-knee amputation with targeted muscle reinnervation. The tissue at the level of below-knee amputation was found to be healthy and free of infection. Warfarin with heparin bridge was initiated postoperatively along with aspirin. The patient was discharged and is due for multispecialty follow-up. The patient consented to the publication of this anonymized case report.

## Discussion

The risk of arterial thrombosis in the setting of gender-affirming hormone therapy has yet to be adequately characterized in the current literature. Age, formulation of gender-affirming hormone therapy, time since therapeutic onset, thrombotic phenotype, personal or family history of thrombosis or coagulopathy, smoking status, obesity, acute illness, and recent surgical intervention can all predispose an individual to a hypercoagulable state, thrombus formation, myocardial infarction, stroke, or limb ischemia.^2^, ^3^, ^4^ Many factors besides estrogen, including our patient’s smoking history, previous suboptimal stenting, and initial issues with medication adherence could all be contributory to her clinical state, and estrogen cannot be considered causative. Despite this, estrogen’s widespread ability to alter platelet activation and aggregation, Von Willebrand factor activity, pro-inflammatory cytokine expression and pathways, fibrinolysis, and plasma concentrations of Factor II, Factor V, Factor VII, Factor X, fibrinogen, Proteins C and Protein S^4^^,^^5^ make understanding the long-term cardiovascular effects of gender-affirming hormone therapy challenging.

Current studies have identified estrogen’s route of administration as having a profound effect on long-term outcomes. Recent studies suggest optimal gender-affirming hormone therapy regimens consist of transdermal estrogen and spironolactone, though no universally accepted guidelines are present to date.^4^^,^^6^ Numerous studies have noted the superior safety profile of transdermal estrogen compared with oral,^2^^,^^4^, ^5^, ^6^, ^7^ likely due to upregulation of proinflammatory cytokines IL-1, IL-6, IL-8, and TNF-alpha,^3^^,^^4^^,^^7^ which lead to increased thrombophilia when taken orally. First pass effect, unique to oral estrogens, also contribute to prothrombic state by increasing synthetic production of Von Willebrand factor, prothrombin, fibrinogen, and Factors VI and VIII, while decreasing anti-thrombin and protein S.^5^^,^^7^ Oral estrogen has also been shown to cause a resistance to activated protein C, promoting thrombosis formation.^5^

Venous thromboembolism is a well-defined and expected risk of estrogen use in the context of cis and transgender populations alike, whether used as contraceptives, hormone therapy in postmenopausal women, or gender-affirming hormone therapy,^5^, ^6^, ^7^ although current literature has yet to characterize the risks and precipitating factors of arteriothrombosis in this population. While discussing this case with vascular surgeons across the region, this phenomenon of arterial thrombosis in a healthy transgender woman otherwise in good cardiovascular health is not unique, and may demonstrate an uncommon, but present, patient population in our field.

The current lack of standardized sex- and gender-concordant data on anticoagulation therapy in transgender women undergoing gender-affirming hormone therapy threatens clinicians’ abilities to provide more complete care for their patients.^4^ Thus, we must rely on data from similar populations, such as postmenopausal female patients taking estrogen or combined hormone replacement therapies. Given the known increased incidence of venous thromboembolism associated with estrogen therapy in this population,^8^^,^^9^ current guidelines recommend avoiding hormone therapy in those with known risk factors for thrombus formation, such as obesity, cancer, or a medical history of venous thromboembolism. At present, it does not appear there is a clear difference in cardiovascular risk when comparing estrogen with combined therapies. Our aim with this case report is stimulate research into the incidence and pathophysiology of thrombosis within native arterial vessels, stents, and bypass grafts within estrogen-naïve individuals on gender affirming hormone therapy, to narrow a growing disparity in the care of transgender women.

## Disclosures

None.
